# Pharmacokinetics and Tissue Distribution of Fluorescently Labeled Neoagarotetraose in Mice

**DOI:** 10.3390/pharmaceutics18060725

**Published:** 2026-06-11

**Authors:** Nan Wu, Chaocheng Wu, Yating Du, Zhuhua Chan, Runying Zeng

**Affiliations:** Technology Innovation Center for Exploitation of Marine Biological Resources, Third Institute of Oceanography, Ministry of Natural Resources, Xiamen 361005, China; wunan@tio.org.cn (N.W.); wuchaocheng@tio.org.cn (C.W.); duyating@tio.org.cn (Y.D.); chan@tio.org.cn (Z.C.)

**Keywords:** neoagarotetraose (NA4), Cy7 labeling, pharmacokinetics, tissue distribution, oral absorption

## Abstract

**Background/Objectives:** Neoagarotetraose (NA4), a marine-derived tetrasaccharide, holds promise as an anti-inflammatory and antioxidant agent; however, its oral bioavailability and systemic exposure mechanisms require elucidation. **Methods:** This study characterizes the biopharmaceutical profile of NA4 after oral and intravenous administration using a validated near-infrared fluorescence method based on covalent conjugation with Cy7. **Results:** Following oral gavage (200 mg/kg), NA4-Cy7 was rapidly absorbed (Tmax: 1.0 h; Cmax: 35.6 mg/L), with prolonged systemic exposure (mean residence time: 13.1 h) and an elimination half-life of 8.9 h. Intravenous administration (25 mg/kg) revealed a low volume of distribution at steady state (Vss: 0.0132 L/kg) and a shorter MRT (4.3 h). Tissue distribution at 24 h showed preferential accumulation in the kidney, liver, and lung, with direct visualization of intact NA4 crossing the intestinal epithelium. **Conclusions:** These findings demonstrate that fluorescently labeled NA4-Cy7 can cross the intestinal epithelial barrier and reach systemic circulation, supporting its potential as an orally active agent with organ-specific targeting properties.

## 1. Introduction

Marine-derived oligosaccharides have emerged as promising bioactive agents for pharmaceutical and nutraceutical applications, exhibiting diverse pharmacological properties including anti-inflammatory, antioxidant, and immunomodulatory activities with potential for systemic therapeutic intervention [1,2,3,4,5]. Neoagaro-oligosaccharides (NAOs), obtained via enzymatic hydrolysis of agar from marine red algae, represent a novel class of structurally defined carbohydrates with low degrees of polymerization and excellent physicochemical stability [6,7]. Neoagarotetraose (NA4), a characteristic tetrasaccharide composed of two neoagarobiose units featuring alternating β-1,4- and α-1,3-glycosidic linkages between D-galactose and 3,6-anhydro-L-galactose residues, exhibits notable antioxidant, anti-inflammatory, and metabolic regulatory activities in macrophage cultures and rodent disease models [8,9,10,11].

Despite the established bioactivity profile, the translation of NA4 from a marine-derived compound to an orally active therapeutic agent with predictable systemic exposure faces critical biopharmaceutical challenges. The current mechanistic paradigm predominantly attributes NA4’s systemic benefits to indirect gut microbiota modulation, while the fraction of intestinal absorption, plasma protein binding, and organ-specific disposition of the intact molecule remain undefined [12,13,14,15]. This knowledge gap significantly limits the rational design of oral formulations and the prediction of dose-exposure relationships—fundamental determinants for establishing efficacious dosing regimens in systemic disease models.

The key to investigating the distribution, metabolism, and action of a compound in vivo lies in establishing a sensitive and reliable tracing methodology. Currently available labeling techniques include fluorescent labeling, isotopic labeling, and immunological methods [16]. Among these, fluorescent labeling is widely employed owing to its operational convenience and high safety profile. Commonly used fluorescent probes include FITC (fluorescein isothiocyanate), TRITC (tetramethylrhodamine isothiocyanate), and near-infrared dyes such as the Cy series. Although FITC offers high quantum yield and mature labeling protocols, its excitation (~488 nm) and emission (~520 nm) wavelengths lie in the visible light region, resulting in limited tissue penetration depth and susceptibility to autofluorescence interference from biological tissues [17]. In contrast, the near-infrared fluorescent probe Cy7 possesses excitation and emission wavelengths at approximately 750 nm and 780 nm, respectively, providing superior tissue penetration, minimal background autofluorescence, and enhanced signal-to-noise ratios, making it particularly suitable for in vivo imaging and deep tissue distribution studies [18,19].

This study employs near-infrared (NIR) fluorescent labeling with Cy7 to establish and validate a sensitive, quantitative bioanalytical methodology for tracking the intestinal absorption and systemic disposition of low-molecular-weight oligosaccharides. We present the synthesis and physicochemical characterization of the NA4-Cy7 conjugate, rigorous validation of a fluorometric bioanalytical method with defined sensitivity and precision, and comprehensive pharmacokinetic profiling following intragastric and intravenous administration in mice. By delineating the absorption kinetics, tissue distribution patterns and organ-specific retention characteristics, this work provides mechanistic insights into the oral bioavailability of NA4 beyond exclusive gut microbiota-mediated mechanisms. These findings establish a biopharmaceutical foundation for developing structurally defined marine-derived neoagaro-oligosaccharides as orally active therapeutic agents with predictable systemic exposure and organ-selective delivery potential.

## 2. Materials and Methods

### 2.1. Drugs and Reagents

NA4 was prepared according to the previous method [20,21]. Coupling reagents including 1-hydroxybenzotriazole (HOBT, purity ≥ 97%), N,N′-diisopropylcarbodiimide (DIC, purity ≥ 99%), and 4-dimethylaminopyridine (DMAP, purity ≥ 99%) were purchased from Merck KGaA (Darmstadt, Germany). Dimethyl sulfoxide (DMSO, chromatographic grade), methanol (chromatographic grade), and diethyl ether (analytical grade) were obtained from Shanghai Aladdin Biochemical Technology Co., Ltd. (Shanghai, China). All salts used for buffer preparation were of analytical grade. Ultrapure water was used throughout all experiments.

### 2.2. NA4-Cy7 Synthetic Procedure

NA4 (30.0 mg) was accurately weighed and dissolved in 3 mL of anhydrous dimethyl sulfoxide (DMSO) with stirring until complete dissolution. To this solution, CY7-COOH (1.0 equivalent), HOBT (3.0 equivalents), DIC (10.0 equivalents), and DMAP (0.2 equivalents) were sequentially added. The reaction mixture was stirred at room temperature (25 °C) under dark conditions for 6 h.

**Figure 1:**
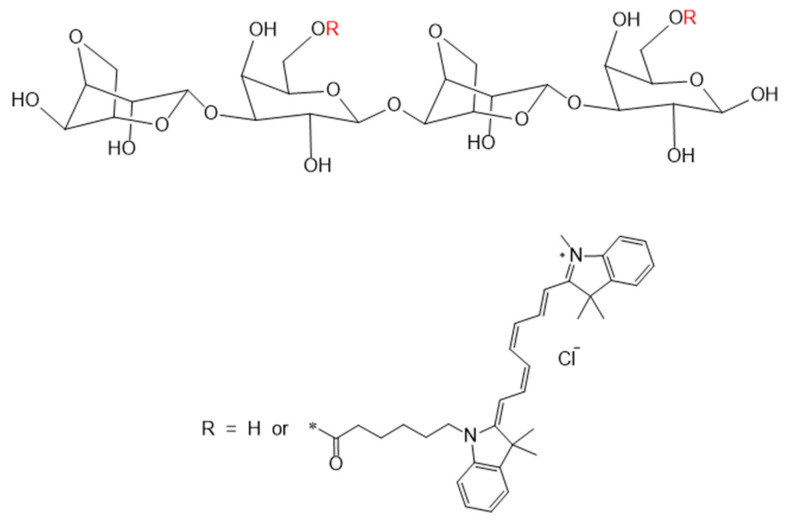
Schematic diagram of the structure of NA4-Cy7.

Upon completion of the reaction, the mixture was evaporated under reduced pressure at 40 °C to remove the majority of DMSO. The resulting concentrate was reconstituted with an appropriate amount of methanol, and this solution was then slowly added dropwise into a large volume of vigorously stirred ice-cold diethyl ether, upon which solid precipitation was observed. The mixture was centrifuged at 4 °C, 12,000× *g* for 10 min, and the precipitate was collected. This “methanol reconstitution–ice ether precipitation” cycle was repeated three times to thoroughly remove unreacted dye, condensing agents, and by-products. The final solid product was vacuum-dried to obtain the NA4-Cy7 conjugate, which was stored at −20 °C protected from light until use. The chemical structure of NA4-Cy7 is illustrated in Figure 1.

### 2.3. UV–Vis and Fluorescence Spectroscopy Analysis

NA4 and NA4-Cy7 were each prepared as 0.1 mg/mL aqueous solutions and scanned using a UV–Vis spectrophotometer over the wavelength range of 400–900 nm.

### 2.4. Determination of Fluorescence Substitution Degree

Cy7-NH_2_ reference standard was accurately weighed and dissolved in PBS to prepare a series of standard solutions at concentrations of 0.1, 0.5, 1.0, 2.0, and 5.0 mg/L. Using PBS as a blank control, fluorescence intensity was measured at an excitation wavelength of 750 nm, emission wavelength of 780 nm, and slit width of 5 nm to establish a standard curve.

### 2.5. Animal Administration and In Vivo Fluorescence Imaging

#### 2.5.1. Animals

Experimental animals were SPF-grade BALB/c-nu mice (male, 6–8 weeks old, 18–22 g), supplied by Fuzhou Wushi Experimental Animal Co., Ltd. (Fuzhou, China). Animals were housed under controlled conditions (22 ± 2 °C, 55 ± 5% humidity, 12 h light/dark cycle) with ad libitum access to standard chow and water. All experimental protocols were approved by the Laboratory Animal Management and Welfare Ethical Review Committee of the Third Institute of Oceanography (Approval No. TIO-IACUC-07-2025).

BALB/c-nu (nude) mice were selected for this study because their hairless phenotype minimizes light scattering and autofluorescence interference during whole-body near-infrared fluorescence imaging, providing superior signal-to-noise ratios compared to furred strains. This strain is commonly used for optical imaging studies and has previously been characterized in our laboratory for baseline fluorescence levels. Although these mice are T-cell deficient, the acute pharmacokinetic and tissue distribution experiments (24 h) are not dependent on adaptive immune responses.

#### 2.5.2. Experimental Design

Mice were randomly allocated to treatment groups (ig or iv) using a computer-generated randomization sequence (Excel RAND function). Sample size (n = 6 per group) was determined based on pilot data (expected Cmax 30 ± 10 mg/L, SD) to provide 80% power to detect a 30% difference between administration routes at α = 0.05 (G*Power 3.1). In vivo imaging analysis was performed by a researcher blinded to treatment allocation to minimize bias.

#### 2.5.3. Formulation Composition

NA4-Cy7 was dissolved in sterile phosphate-buffered saline (PBS, pH 7.4) immediately before administration. The dosing volume was 10 mL/kg body weight for all animals. For the intragastric (ig) group (200 mg/kg), the formulation concentration was 20 mg/mL; for the intravenous (iv) group (25 mg/kg), the formulation concentration was 2.5 mg/mL. The iv formulation was filtered through a 0.22 μm sterile filter prior to injection. All formulations were protected from light and used within 30 min of preparation.

#### 2.5.4. Dose Selection Rationale

The oral dose of 200 mg/kg and the intravenous dose of 25 mg/kg were selected based on the following considerations: (i) previous in vivo efficacy studies on neoagaro-oligosaccharides, including NA4, have commonly used oral doses ranging from 100 to 500 mg/kg to achieve significant anti-inflammatory or gut microbiota-modulating effects [13,14,22]; (ii) due to the relatively low fluorescence substitution degree of NA4-Cy7 (0.86%), a sufficiently high administered mass was necessary to maintain detectable fluorescence signals above the LOQ (0.1 mg/L) at later time points (e.g., 24 h); (iii) based on the observed plasma concentration–time profiles and the fact that the oral Cmax (35.6 mg/L, ~56 μM) is far below the reported Km values of intestinal sugar transporters (in the mM range), we do not expect saturation of absorption or elimination pathways at this dose. The measured concentrations all fell within the validated linear range (0.5–200 mg/L), further supporting the absence of saturation. Formal dose-proportionality studies will be conducted in future investigations.

### 2.6. Establishment of Quantitative Analytical Method for NA4-Cy7 in Mouse Plasma and Tissues

#### 2.6.1. Standard Curve Preparation and Validation Range

Fluorescence intensity was measured using a Shimadzu RF-6000 spectrofluorophotometer (Shimadzu Corporation, Kyoto, Japan) with excitation at 746 nm, emission at 772 nm, and slit widths of 5.0 nm.

Standard samples were prepared covering 0.5–200 mg/L (low: 0.5, 1, 5; medium: 10, 50, 100; high: 200 mg/L) to encompass the diluted sample concentrations following iv administration (plasma and tissue homogenates were diluted 1:10 with PBS prior to detection; see Section 2.7). Limit of Detection (LOD) and Limit of Quantification (LOQ) were determined based on signal-to-noise ratios (S/N ≥ 3 for LOD, S/N ≥ 10 for LOQ), yielding LOD of 0.03 mg/L and LOQ of 0.1 mg/L. The validated linear range (LLOQ–ULOQ) for all biological matrices was established as 0.5–200 mg/L, within which linear regression using weighted least squares (1/X^2^) demonstrated excellent correlation (R^2^ > 0.999).

#### 2.6.2. Precision and Accuracy Evaluation

Low, medium, and high concentration levels (0.5, 5, and 25 mg/L) of NA4-Cy7 were spiked into blank plasma and tissue homogenates (heart, liver, spleen, lung, kidney, stomach, small intestine, brain). Six replicates per concentration were analyzed over three consecutive days. Intra-day and inter-day precision (RSD) and accuracy (relative recovery) were calculated.

#### 2.6.3. Stability Evaluation

Stability was assessed under three conditions: ambient temperature (24 h), 4 °C (7 days), and −20 °C (30 days). QC samples at three concentrations were analyzed, and RSD values were calculated.

### 2.7. Pharmacokinetics and Tissue Distribution of NA4-Cy7

Blood samples (~100 μL) were collected from the tail vein of mice in both the ig and iv groups at predetermined time points (0.5, 1, 2, 4, 8, 12, and 24 h post-dose) and placed in heparin-pretreated centrifuge tubes. Plasma was obtained by centrifugation at 1500× *g* for 15 min. A 50 μL aliquot of plasma was mixed with 450 μL of PBS for fluorescence intensity measurement.

At 24 h post-administration, mice were euthanized and the heart, liver, spleen, lung, kidney, brain, and small intestine were rapidly harvested. Tissues were rinsed with ice-cold PBS, dried with filter paper, and accurately weighed. Tissue homogenates were prepared by adding 9 volumes of PBS (*v*/*w*) and then centrifuged at 1500× *g* for 15 min. The supernatants were appropriately diluted prior to fluorescence intensity determination. Concurrently, liver and kidney specimens were fixed in 4% paraformaldehyde, subjected to sucrose gradient dehydration, embedded in OCT compound, and sectioned for cryosectioning. The distribution of NA4-Cy7 was visualized under confocal laser scanning microscopy.

### 2.8. Data Analysis

Pharmacokinetic parameters were calculated using the non-compartmental model in DAS 3.0 software. The calculated parameters included half-life (t_1_/_2_), area under the concentration-time curve (AUC_0–t_ and AUC_0–∞_), mean residence time (MRT), and volume of distribution. For intragastric administration, peak concentration (Cmax) and time to peak concentration (Tmax) were reported, and the apparent volume of distribution based on the terminal phase was calculated as Vz/F = Dose/(λ_z × AUC_0–∞_), where λ_z is the terminal elimination rate constant. For intravenous administration, the initial concentration at time zero (C_0_) was estimated by log-linear back-extrapolation of the first two measured concentrations (0.5 h and 1 h); the elimination-phase volume of distribution was calculated as Vz = Dose/(λ_z × AUC_0–∞_), and the steady-state volume of distribution (Vss) was derived from standard non-compartmental formulas. Because bioavailability F = 1 after intravenous dosing, Vz/F reduces to Vz.

All data were expressed as mean ± standard deviation (SD). Statistical analysis was performed using SPSS 26.0 software with one-way analysis of variance (ANOVA); *p* < 0.05 was considered statistically significant. In vivo imaging data were analyzed with Living Image 4.5 software, and tissue section images were processed using ImageJ 1.53 software.

## 3. Results

### 3.1. Preparation and Characterization of NA4-Cy7

**Figure 2:**
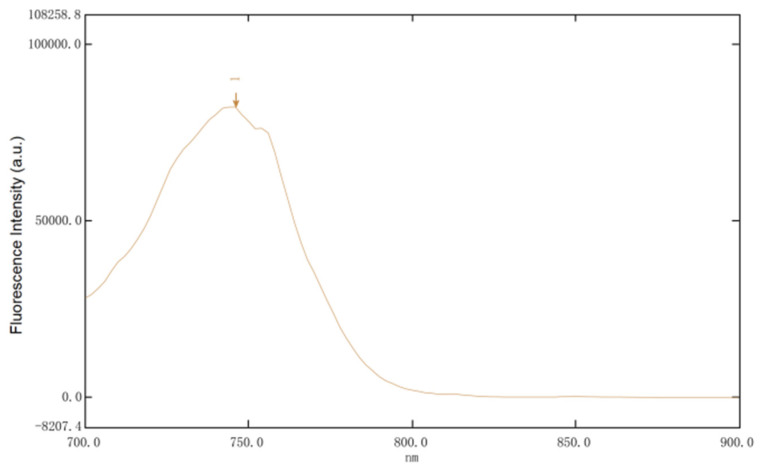
UV-Vis absorption spectra of NA4 and NA4-Cy7.

For spectroscopic characterization, the NA4-Cy7 conjugate was dissolved in ultrapure water to prepare a 1 mg/mL solution. As shown in Figure 2, UV–Vis absorption spectroscopy revealed that, compared with unlabeled NA4, NA4-Cy7 exhibited a distinct absorption peak at 746 nm, corresponding to the characteristic maximum absorption of the Cy7 fluorophore. This result confirms the successful covalent conjugation of NA4 with Cy7. Fluorescence spectroscopy further demonstrated (Table 1) that the conjugate displayed a maximum excitation wavelength (λex) of 746 nm and a maximum emission wavelength (λem) of 772 nm. Under the selected acquisition parameters, the maximum fluorescence intensity reached 82,197, indicating strong near-infrared fluorescence signals suitable for in vivo tracking.

### 3.2. Fluorescence Substitution Degree of NA4-Cy7

**Figure 3:**
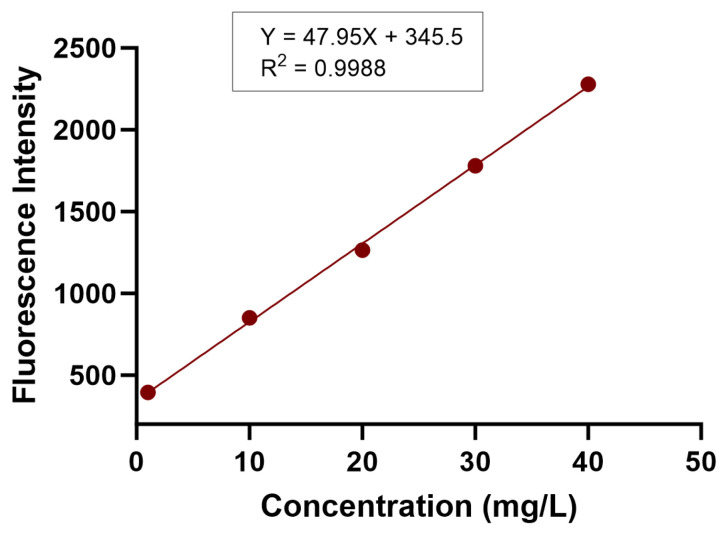
Standard curve for determination of fluorescence substitution degree using Cy7-NH_2_ reference standard in PBS (0.1–5.0 mg/L). This calibration is independent of the biological matrix quantification used for pharmacokinetic analysis. N = 6.

The fluorescence substitution degree was determined using fluorescence spectrophotometry. Serial Cy7 standard solutions were measured under the following conditions: excitation wavelength 746 nm, emission wavelength 772 nm, and excitation/emission bandwidths both set at 5.0 nm. The resulting standard curve was described by the equation Y = 47.95X + 345.5, with a correlation coefficient of R^2^ = 0.9988 (Figure 3). Under identical conditions, the NA4-Cy7 sample solution (1 mg/mL) was diluted to an appropriate concentration, yielding a fluorescence intensity of 757.8. Substituting this value into the standard curve equation, the fluorescence substitution degree was calculated to be 0.86%, corresponding to 0.86 μg of Cy7 dye conjugated per 100 μg of NA4.

### 3.3. Quantitative Analytical Method for NA4-Cy7 in Plasma and Tissue Homogenates

Standard curves for NA4-Cy7 in plasma and various tissue homogenates (heart, liver, spleen, lung, kidney, stomach, small intestine, brain) exhibited good linearity over the concentration range of 0.5–200 mg/L, with correlation coefficients (R^2^) exceeding 0.99 for all matrices (Table 2). The limit of detection (LOD) was 0.03 mg/L, and the limit of quantification (LOQ) was 0.1 mg/L.

#### 3.3.1. Precision Test

As summarized in Table 3, the intra-day precision of NA4-Cy7 ranged from 3.25% to 8.76% RSD, and the inter-day precision ranged from 4.83% to 10.24% RSD at low (0.5 mg/L), medium (5 mg/L), and high (25 mg/L) concentration levels. All values were within the acceptable limit of RSD < 15%, indicating satisfactory precision of the established method across all tested matrices.

#### 3.3.2. Stability Test

The stability of NA4-Cy7 in plasma and tissue homogenates was evaluated under three storage conditions (Table 4). The RSD values remained within 4.37–9.85% after 24 h at room temperature, 5.26–11.34% after 7 days at 4 °C, and 6.72–12.58% after 30 days at −20 °C, demonstrating good stability of the conjugate under the tested conditions.

#### 3.3.3. Recovery Test

The accuracy of the method was assessed by spiking NA4-Cy7 at three concentration levels into blank biological matrices. As shown in Table 5, the mean recoveries ranged from 94.6% to 107.8% across plasma and all tissue homogenates, with associated RSD values below 13.5% for all matrices. These results confirm the reliability of the established quantitative method.

### 3.4. In Vivo Fluorescence Imaging Analysis

**Figure 4:**
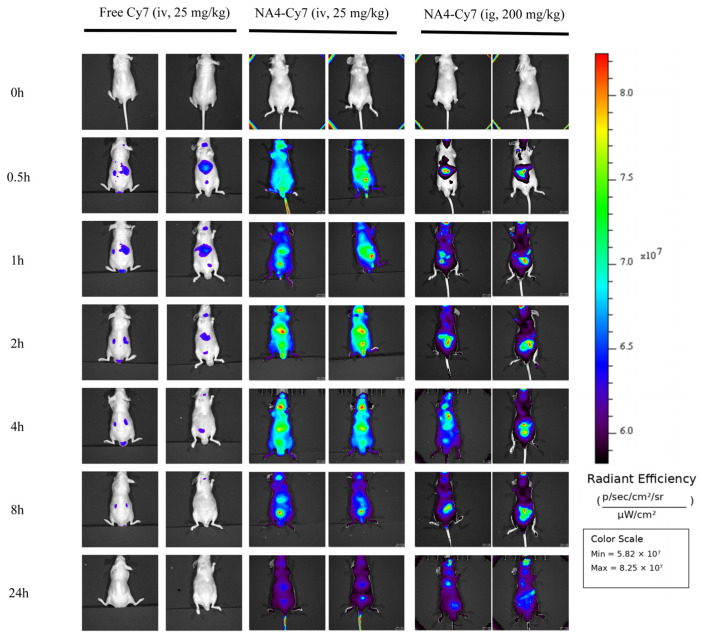
In vivo whole-body fluorescence imaging of NA4-Cy7 in BALB/c-nu mice. Mice received a single administration of NA4-Cy7 via iv (25 mg/kg) or ig (200 mg/kg). Whole-body fluorescence images were acquired at 0, 0.5, 1, 2, 4, 8, and 24 h post-administration using a small animal in vivo imaging system. N = 6.

Whole-body fluorescence images of mice following a single administration of NA4-Cy7 are presented in Figure 4. In the intravenous (iv) group (25 mg/kg), high fluorescence signals were observed as early as 0.5 h post-dose, peaked at 1 h, and remained at an intensity of approximately 6.0 × 10^7^ at 24 h. In the intragastric (ig) group (200 mg/kg), fluorescence signals were lower at 0.5 h, reached a peak at 4 h, and then gradually declined. Compared with the iv route, the ig route resulted in delayed absorption but better signal maintenance at later time points. Both administration routes confirmed that NA4-Cy7 possessed favorable fluorescence stability and persistent distribution characteristics in vivo.

### 3.5. Concentration-Time Profiles

**Figure 5:**
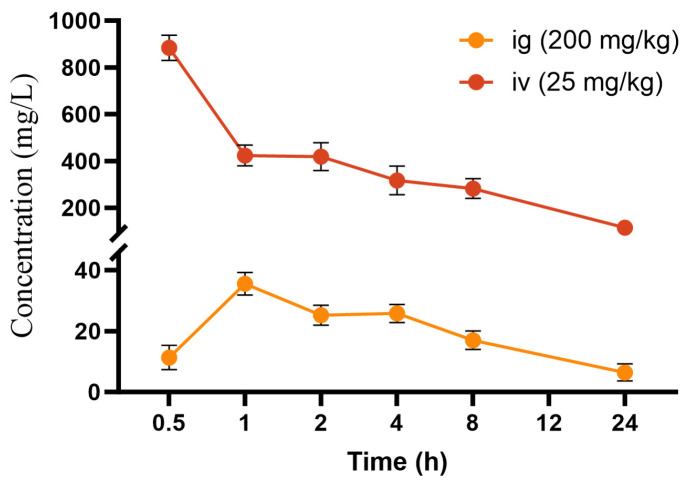
Mean plasma concentration-time profiles of NA4-Cy7 in mice. Mice received a single dose of NA4-Cy7 via ig (200 mg/kg) or iv (25 mg/kg). Plasma concentrations were determined at 0.5, 1, 2, 4, 8, 12, and 24 h post-administration using a fluorescence-based quantitative method. Data are presented as mean ± SD. N = 6.

The mean plasma concentration–time profiles of NA4-Cy7 following single ig (200 mg/kg) and iv (25 mg/kg) administrations in mice are illustrated in Figure 5. After iv administration, the plasma concentration peaked at the first sampling point (0.5 h), followed by a typical biphasic decline consisting of an initial rapid distribution phase and a subsequent slower elimination phase, consistent with two-compartment model characteristics. After ig administration, the plasma concentration peaked at 1 h, indicating rapid gastrointestinal absorption. Thereafter, the concentration declined slowly, with detectable drug levels remaining significantly above baseline at 24 h post-dose, suggesting either slow elimination or potential enterohepatic circulation of NA4-Cy7 in vivo.

### 3.6. Pharmacokinetic Parameters

The main pharmacokinetic parameters of NA4-Cy7 after ig and iv administration are summarized in Table 6. Following oral administration, NA4-Cy7 was detectable in the blood, reaching a Cmax of 35.6 mg/L at a Tmax of 1.0 h. Vz/F was low, indicating that even after accounting for oral bioavailability, the systemic distribution of NA4-Cy7 remained relatively limited. The MRT was 13.1 h, suggesting prolonged circulation characteristics.

Following iv administration, NA4-Cy7 rapidly entered the systemic circulation and exhibited high systemic exposure levels. Vss was low (0.0132 L/kg), suggesting that NA4-Cy7 was primarily confined within the vascular compartment rather than extensively distributing into peripheral tissues. The MRT was 4.3 h, which is notably longer compared with typical oligosaccharide molecules, indicating prolonged in vivo retention. For the intravenous route, C_0_ was 1228 mg/L, and Vz was 0.0380 L/kg (Table 6).

### 3.7. Tissue Distribution Characteristics

**Figure 6:**
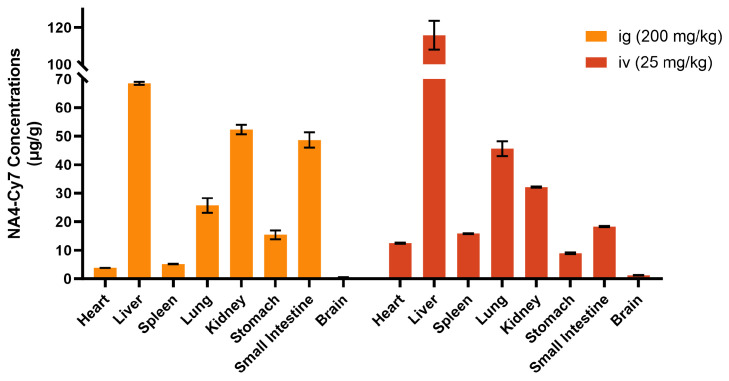
Tissue distribution of NA4-Cy7 in mice at 24 h post-administration. Mice received a single dose of NA4-Cy7 via ig (200 mg/kg) or iv (25 mg/kg). At 24 h, major organs were harvested and drug concentrations were quantified using a fluorescence-based method. Data are presented as mean ± SD. N = 6.

At 24 h post-administration, animals were euthanized, and the distribution of NA4-Cy7 in various tissues was determined (Figure 6). Both administration routes yielded detectable drug concentrations in all examined tissues. For the ig group, the tissue concentrations ranked as follows: liver > kidney > small intestine > lung > stomach > spleen > heart > brain. For the iv group, the distribution pattern was: liver > lung > kidney > small intestine > spleen > heart > stomach > brain. Notably, trace amounts of NA4-Cy7 were detected in brain tissue following both routes.

### 3.8. Distribution Characteristics in Major Organs

**Figure 7:**
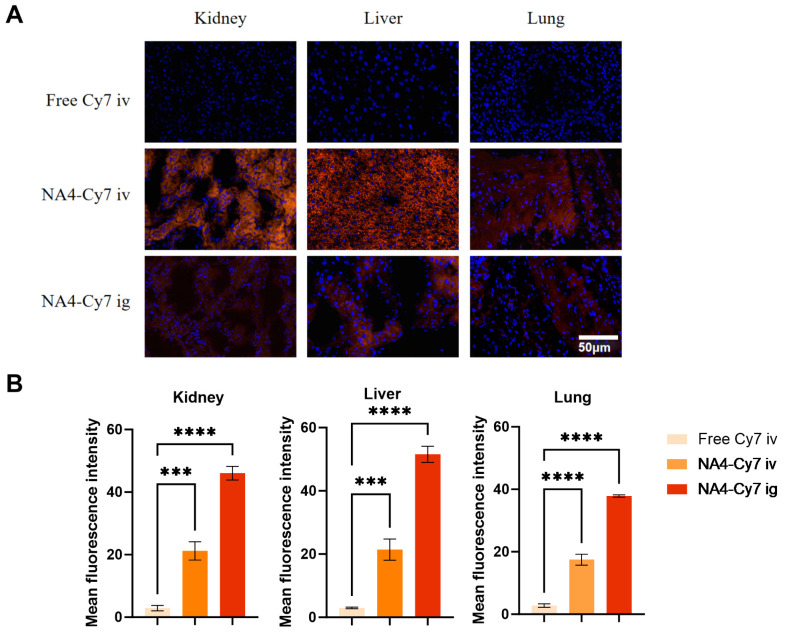
Distribution characteristics of NA4-Cy7 in major organs (kidney, liver, lung) at 24 h post-administration. (**A**) Representative fluorescence images for the free Cy7 iv (25 mg/kg, blank control), NA4-Cy7 iv (25 mg/kg), and NA4-Cy7 ig (200 mg/kg) groups. The free Cy7 iv group shows intrinsic fluorescence of the unbound dye. (**B**) Quantitative analysis of Cy7 fluorescence intensity. *** *p* < 0.001, **** *p* < 0.0001 vs. the free Cy7 iv group. Data are presented as mean ± SD. N = 6.

The distribution characteristics of NA4-Cy7 in the kidney, liver, and lung were further analyzed (Figure 7). In kidney tissue, the fluorescence intensity in the cortical region was significantly higher than that in the medullary region, suggesting that NA4-Cy7 is primarily excreted via glomerular filtration and tubular secretion. In liver tissue, fluorescence signals were uniformly distributed throughout the hepatic lobules, with slight enrichment in the peri-central venous regions. In lung tissue, fluorescence was primarily localized in the alveolar septa and capillary regions, indicating that NA4-Cy7 can cross the alveolar–capillary barrier. These distribution features provide important insights into the metabolic pathways and potential targeted effects of NA4 in vivo. Quantitative analysis of fluorescence intensity (Figure 7B) confirmed that both NA4-Cy7 iv and ig groups exhibited significantly higher signals in the kidney, liver, and lung compared to the free Cy7 iv control group.

## 4. Discussion

Research on the in vivo behavior of oligosaccharides has long been constrained by detection sensitivity and tissue penetration depth [23]. Conventional FITC labeling, with emission in the visible region (~520 nm), is susceptible to autofluorescence interference from tissues and suffers from limited penetration depth, making it difficult to capture low-abundance signals in blood [24]. Consequently, most studies have only observed fluorescence accumulation in the gastrointestinal tract and liver, leading to the inference that oligosaccharides primarily exert effects indirectly via gut microbiota [25,26]. In contrast, the Cy7 near-infrared probe adopted in this study (excitation/emission: 746/772 nm), leveraging deeper tissue penetration and higher signal-to-noise ratios, achieved, for the first time, full-course visualization of NA4 in mice, directly capturing its dynamic process of crossing the intestinal barrier into the bloodstream and distributing to distal organs such as the liver, lung, and kidney. This technical breakthrough not only compensates for the shortcomings of FITC in in vivo quantification but also provides a methodological paradigm that can be referenced for investigating the systemic mechanisms of marine-derived oligosaccharides.

Previous studies have predominantly explored the functions of NAOs from the gut microbiome perspective—for instance, employing 16S rRNA sequencing and metabolomics to demonstrate their ability to modulate microbial communities and promote short-chain fatty acid production, thereby ameliorating obesity or colitis [14,22]. However, these investigations did not directly track the absorption and circulation of the oligosaccharides themselves, leaving the question of whether they exert systemic effects as intact molecules largely unsubstantiated. This study demonstrates, for the first time, that plasma concentration of NA4 peaked at 1 h following oral administration, with the parent compound still detectable in tissues at 24 h post-dose. This unequivocally indicates that NA4 can bypass complete degradation by gut microbiota and be directly absorbed into the bloodstream in intact or near-intact molecular form, challenging the conventional paradigm that marine oligosaccharides act exclusively through indirect microbial mechanisms. This finding provides direct material evidence for explaining the systemic bioactivity of NAOs and expands their mechanism of action from a singular “gut microbiota modulation” to “systemic effects following direct absorption.”

NA4 exhibited significant route-dependent differences in MRT: the ig group was markedly higher than the iv group, suggesting that oral absorption represents the rate-limiting step. After oral administration, the drug entered circulation slowly and continuously, creating an “extended absorption window effect” that avoided sharp fluctuations in plasma concentration [27]. IV administration, conversely, displayed a “high exposure–low distribution–slow clearance” pattern: the Vss of only 0.0132 L/kg suggests that NA4 rapidly binds to plasma proteins (e.g., albumin) or forms molecular aggregates upon entering the bloodstream and is confined within the vascular compartment, thereby delays hepatic and renal clearance. This mechanism explains its prolonged terminal half-life and also indicates that the stability of NA4 in blood circulation provides a temporal window for targeted tissue accumulation.

At 24 h post-dose, NA4 exhibited the highest concentrations in the kidney and liver, followed by the lung and spleen. The iv group demonstrated pronounced liver-targeting properties, suggesting that the asialoglycoprotein receptor on hepatocytes may mediate efficient uptake. The ig group, in contrast, showed a triple-distribution pattern in liver–lung–kidney: renal enrichment confirmed its excretion via glomerular filtration and tubular secretion; high distribution in lung tissue may be associated with alveolar-capillary permeability and may even hint at the existence of direct oligosaccharide trafficking along the “gut–lung axis” [28,29,30], offering clues regarding the potential of NA4 in respiratory disease intervention. Brain tissue concentrations in both routes were at the lower limit of detection, indicating its limited ability to penetrate the intact blood–brain barrier.

In recent years, fluorescent labeling and tracing have also been performed on chitosan, hyaluronic acid, and fucoidan. Similarities lie in the use of FITC or Cy series dyes and the observation that the liver and kidney serve as primary distribution organs, suggesting that hepato-renal metabolism constitutes a common metabolic fate for carbohydrate-based substances [31,32,33,34]. However, this study demonstrates a stark contrast with existing reports at three core levels: molecular weight selection, labeling strategy, and detection sensitivity. At the molecular weight dimension, this study employed NA4 with a defined degree of polymerization (DP = 4), whereas most polysaccharide studies utilize high-molecular-weight (>10 kDa) samples with extremely low absorption rates, allowing only local retention to be observed [35,36,37,38]. Regarding labeling strategy, stable covalent labeling was achieved through carboxyl-hydroxyl condensation in this study, whereas some investigations employ unstable physical encapsulation, leading to dye dissociation and false-positive signals [39]. In terms of detection sensitivity, Cy7 near-infrared labeling enabled this study to quantitatively detect mg/L-level concentrations in blood, whereas FITC-labeled studies often suffer from background interference and can only provide qualitative observations. These differences highlight the unique advantages of NA4 as a medium-molecular-weight oligosaccharide in oral absorption and systemic distribution.

Notably, some studies have reported anti-inflammatory and immunomodulatory activities of NA4 without examining gut microbiota, resulting in ambiguous mechanistic interpretations. For instance, NAOs significantly reduced inflammatory cytokines in a lipopolysaccharide-induced acute lung injury model; the mechanism was tentatively attributed to “possible microbial regulation” at the time due to limited detection methods. While recent studies have indeed demonstrated that NAOs modulate gut microbiota to alleviate obesity and metabolic syndrome [40], direct evidence for systemic absorption and distal organ effects remained elusive. The present study provides the first pharmacokinetic confirmation that NA4 can cross the intestinal barrier in intact form, reach systemic circulation, and distribute directly to lung tissue, thereby establishing a dual mechanism of action through both gut microbiota modulation and direct systemic effects. Similarly, its hepatoprotective effects in liver disease models can also be attributed to direct cytoprotection following liver-targeted distribution. This study provides the missing pharmacokinetic link for such “mechanistically unclear” oligosaccharide research, establishing a complete chain connecting in vitro activity to in vivo effects through “absorption–distribution.”

Several limitations should be acknowledged. First, our data reflect the NA4-Cy7 conjugate, not native NA4; the Cy7 label alters molecular weight, hydrophobicity, and potentially pharmacokinetics. Second, fluorescence cannot distinguish intact conjugate from degradation products or free dye. An intragastric free Cy7 group was not included due to poor aqueous solubility, but the intravenous free Cy7 control (Figure 4) ruled out non-specific dye signals. Third, only single doses were tested; although saturation is unlikely (first-order profile, Cmax far below transporter Km, all concentrations within validated linear range), dose-escalation studies are warranted. Future work should employ LC-MS/MS to quantify native NA4, investigate organ-specific mechanisms (e.g., liver uptake, gut–lung axis), and clarify the metabolic fate using mass spectrometry imaging or radiolabeling.

## 5. Conclusions

This study demonstrates that the fluorescently labeled NA4-Cy7 conjugate can cross the intestinal epithelial barrier after oral administration and distributes preferentially to the liver, kidney, and lung in mice, providing direct visualization evidence that a low-molecular-weight neoagaro-oligosaccharide can achieve systemic absorption.

## Figures and Tables

**Table 1:** Fluorescence spectral characterization of the NA4-Cy7 conjugate. N = 6.

Parameter	Value
Excitation maximum (λex)	746 nm
Emission maximum (λem)	772 nm
Maximum fluorescence intensity	82,197
Excitation bandwidth	5.0 nm
Emission bandwidth	5.0 nm
Scan speed	6000 nm/min
Data interval	2.0 nm
Sample concentration	1 mg/mL
Solvent	Ultrapure water

Note: Fluorescence intensity measured at excitation maximum with emission wavelength fixed at 772 nm.

**Table 2:** Standard curve parameters of NA4-Cy7 in plasma and tissue homogenates. N = 6.

Tissue	Linear Equation	Linear Range (mg/L)	R^2^
Plasma	y = 4728.5x + 185.2	0.5–200	0.9995
Heart	y = 4450.3x − 122.7	0.5–200	0.9989
Liver	y = 3981.6x + 98.4	0.5–200	0.9982
Spleen	y = 5124.9x + 205.6	0.5–200	0.9991
Lung	y = 4480.1x − 118.3	0.5–200	0.9985
Kidney	y = 4255.7x + 135.8	0.5–200	0.9997
Stomach	y = 3925.4x + 201.5	0.5–200	0.9993
Small Intestine	y = 5058.2x − 105.4	0.5–200	0.9978
Brain	y = 4632.0x + 125.9	0.5–200	0.9986

**Table 3:** Precision of NA4-Cy7 in plasma and tissue homogenates. N = 6.

Tissue	Concentration mg/L	Intra-Day Measured (mg/L)	Intra-Day Accuracy (%)	Intra-Day RSD (%)	Inter-Day Measured (mg/L)	Inter-Day Accuracy (%)	Inter-Day RSD (%)
Plasma	0.5	0.492 ± 0.057	98.4	11.6	0.492 ± 0.051	98.4	10.3
	5	4.835 ± 0.392	96.7	8.1	4.835 ± 0.445	96.7	9.2
	25	25.120 ± 2.738	100.5	10.9	25.120 ± 2.964	100.5	11.8
Heart	0.5	0.518 ± 0.047	103.6	9	0.518 ± 0.045	103.6	8.6
	5	5.005 ± 0.290	100.1	5.8	5.005 ± 0.225	100.1	4.5
	25	24.805 ± 2.878	99.2	11.6	24.805 ± 2.977	99.2	12
Liver	0.5	0.508 ± 0.028	101.6	5.5	0.508 ± 0.044	101.6	8.7
	5	5.085 ± 0.554	101.7	10.9	5.085 ± 0.447	101.7	8.8
	25	24.850 ± 1.292	99.4	5.2	24.850 ± 1.367	99.4	5.5
Spleen	0.5	0.525 ± 0.062	105	11.8	0.525 ± 0.062	105	11.9
	5	4.935 ± 0.508	98.7	10.3	4.935 ± 0.503	98.7	10.2
	25	25.050 ± 2.330	100.2	9.3	25.050 ± 2.079	100.2	8.3
Lung	0.5	0.503 ± 0.058	100.6	11.6	0.503 ± 0.045	100.6	8.9
	5	4.890 ± 0.289	97.8	5.9	4.890 ± 0.352	97.8	7.2
	25	25.015 ± 2.426	100.1	9.7	25.015 ± 2.251	100.1	9
Kidney	0.5	0.506 ± 0.018	101.2	3.5	0.506 ± 0.059	101.2	11.6
	5	5.002 ± 0.545	100	10.9	5.002 ± 0.600	100	12
	25	24.945 ± 1.596	99.8	6.4	24.945 ± 1.846	99.8	7.4
Stomach	0.5	0.512 ± 0.050	102.4	9.7	0.512 ± 0.032	102.4	6.3
	5	5.025 ± 0.563	100.5	11.2	5.025 ± 0.513	100.5	10.2
	25	25.000 ± 2.550	100	10.2	25.000 ± 3.075	100	12.3
Small Intestine	0.5	0.508 ± 0.044	101.6	8.7	0.508 ± 0.053	101.6	10.5
	5	5.008 ± 0.456	100.2	9.1	5.008 ± 0.431	100.2	8.6
	25	24.945 ± 3.243	99.8	13	24.945 ± 1.896	99.8	7.6
Brain	0.5	0.540 ± 0.062	108	11.4	0.540 ± 0.045	108	8.4
	5	4.935 ± 0.410	98.7	8.3	4.935 ± 0.365	98.7	7.4
	25	25.030 ± 1.327	100.1	5.3	25.030 ± 3.079	100.1	12.3

**Table 4:** Stability of NA4-Cy7 in plasma and tissue homogenates under various storage conditions. N = 6.

Tissue	Conc.	RT 24 h Measured Conc.	RSD (%)	4 °C 7 d Measured Conc.	RSD (%)	−20 °C 30 d Measured Conc.	RSD (%)
Plasma	0.5	0.510 ± 0.042	8.24	0.491 ± 0.055	11.21	0.525 ± 0.036	6.86
	5	4.848 ± 0.259	5.34	4.756 ± 0.417	8.77	5.014 ± 0.420	8.38
	25	25.021 ± 2.975	11.89	25.115 ± 2.711	10.81	24.855 ± 1.608	6.47
Heart	0.5	0.517 ± 0.031	5.99	0.547 ± 0.038	6.95	0.592 ± 0.046	7.77
	5	4.936 ± 0.243	4.92	5.084 ± 0.590	11.61	5.103 ± 0.427	8.37
	25	24.889 ± 3.036	12.19	24.947 ± 1.493	5.99	24.806 ± 1.576	6.34
Liver	0.5	0.493 ± 0.028	5.68	0.527 ± 0.050	9.49	0.484 ± 0.043	8.88
	5	4.871 ± 0.418	8.57	5.009 ± 0.342	6.83	4.788 ± 0.400	8.35
	25	24.869 ± 1.328	5.34	24.956 ± 2.718	10.92	24.953 ± 2.896	11.61
Spleen	0.5	0.520 ± 0.057	10.96	0.544 ± 0.070	12.87	0.503 ± 0.034	6.76
	5	5.084 ± 0.434	8.54	4.849 ± 0.264	5.44	4.992 ± 0.620	12.41
	25	25.148 ± 2.542	10.10	25.129 ± 2.712	10.85	25.231 ± 2.175	8.62
Lung	0.5	0.502 ± 0.048	9.56	0.503 ± 0.049	9.74	0.503 ± 0.048	9.54
	5	4.830 ± 0.546	11.30	4.813 ± 0.558	11.59	4.913 ± 0.369	7.51
	25	24.997 ± 3.075	12.31	24.848 ± 1.926	7.74	24.922 ± 1.179	4.73
Kidney	0.5	0.503 ± 0.027	5.37	0.525 ± 0.057	10.85	0.512 ± 0.065	12.69
	5	4.942 ± 0.350	7.08	4.933 ± 0.269	5.44	5.026 ± 0.538	10.72
	25	24.920 ± 2.764	11.09	24.852 ± 2.478	9.97	25.004 ± 2.877	11.53
Stomach	0.5	0.507 ± 0.061	12.03	0.507 ± 0.061	12.03	0.524 ± 0.056	10.69
	5	5.108 ± 0.582	11.40	5.190 ± 0.550	10.59	5.117 ± 0.301	5.88
	25	24.905 ± 2.474	9.93	25.161 ± 1.756	6.98	24.989 ± 2.225	8.91
Small Intestine	0.5	0.511 ± 0.046	9.00	0.514 ± 0.046	8.95	0.518 ± 0.030	5.79
	5	4.926 ± 0.534	10.84	5.090 ± 0.530	10.41	4.760 ± 0.478	10.04
	25	24.813 ± 2.279	9.18	24.939 ± 2.942	11.80	24.980 ± 2.975	11.91
Brain	0.5	0.520 ± 0.063	12.12	0.543 ± 0.033	6.08	0.528 ± 0.041	7.77
	5	4.715 ± 0.252	5.35	5.014 ± 0.299	5.96	4.882 ± 0.297	6.08
	25	24.959 ± 2.228	8.93	24.944 ± 3.012	12.08	25.043 ± 1.435	5.73

**Table 5:** Recovery of NA4-Cy7 from spiked plasma and tissue homogenates. N = 6.

Tissue	Spiked Conc.	Measured Conc.	Recovery (%)	RSD (%)
Plasma	0.5	0.489 ± 0.026	97.8	5.32
	5	4.872 ± 0.411	97.4	8.44
	25	25.210 ± 0.665	100.8	2.64
Heart	0.5	0.525 ± 0.058	105.0	11.05
	5	4.995 ± 0.353	99.9	7.07
	25	24.750 ± 0.433	99.0	1.75
Liver	0.5	0.516 ± 0.060	103.2	11.63
	5	5.012 ± 0.290	100.2	5.79
	25	24.905 ± 0.618	99.6	2.48
Spleen	0.5	0.528 ± 0.056	105.6	10.61
	5	4.925 ± 0.219	98.5	4.45
	25	25.195 ± 0.310	100.8	1.23
Lung	0.5	0.495 ± 0.052	99.0	10.51
	5	4.730 ± 0.277	94.6	5.86
	25	24.905 ± 0.550	99.6	2.21
Kidney	0.5	0.518 ± 0.045	103.6	8.69
	5	5.018 ± 0.061	100.4	1.22
	25	24.935 ± 0.434	99.7	1.74
Stomach	0.5	0.513 ± 0.053	102.6	10.33
	5	5.025 ± 0.650	100.5	12.94
	25	25.105 ± 0.587	100.4	2.34
Small Intestine	0.5	0.523 ± 0.070	104.6	13.38
	5	5.165 ± 0.272	103.3	5.27
	25	25.025 ± 0.641	100.1	2.56
Brain	0.5	0.552 ± 0.055	110.4	9.96
	5	4.840 ± 0.421	96.8	8.70
	25	25.035 ± 0.433	100.1	1.73

**Table 6:** Main pharmacokinetic parameters of NA4-Cy7 in mice after a single intragastric (ig) or intravenous (iv) administration. N = 6.

Pharmacokinetic Parameter	ig (200 mg/kg)	iv (25 mg/kg)
AUC (0–24) h·mg/L	213.5	5487.9
AUC (0–∞) h·mg/L	233.8	5512.6
Cmax (mg/L)	35.6	—
C_0_ (mg/L)	—	1228
T_max_ (h)	1.0	—
T_1_/_2_ (h)	8.9	5.8
Vz/F (ig) or Vz (iv) (L/kg)	69.2	0.0380
MRT (h)	13.1	4.3
V_ss_ (L/kg)	—	0.0132

## Data Availability

The original contributions presented in this study are included in the article. Further inquiries can be directed to the corresponding author.

## Abbreviations

The following abbreviations are used in this manuscript:

NA4	Neoagarotetraose
FITC	fluorescein isothiocyanate
TRITC	tetramethylrhodamine isothiocyanate
NIR	near-infrared
HOBT	1-hydroxybenzotriazole
DIC	N,N′-diisopropylcarbodiimide
DMSO	anhydrous dimethyl sulfoxide
LOQ	Limit of Quantification
